# Deptor: not only a mTOR inhibitor

**DOI:** 10.1186/s13046-016-0484-y

**Published:** 2017-01-13

**Authors:** Valeria Catena, Maurizio Fanciulli

**Affiliations:** SAFU, Department of Research, Advanced Diagnostics, and Technological Innovation, Translational Research Area, Regina Elena National Cancer Institute, 00144 Rome, Italy

**Keywords:** Deptor, mTOR, ER homeostasis, Cancer, Cancer therapy

## Abstract

Deptor is an important protein that belongs to the mTORC1 and mTORC2 complexes, able to interact with mTOR and to inhibit its kinase activity. As a natural mTOR inhibitor, Deptor is involved in several molecular pathways, such as cell growth, apoptosis, autophagy and ER stress response. For this reason, Deptor seems to play an important role in controlling cellular homeostasis. Despite several recent insights characterizing Deptor functions and regulation, its complete role within cells has not yet been completely clarified. Indeed, quite recently, Deptor has been associated with chromatin, and it has been demonstrated having a role in transcriptional regulation, controlling in such way endoplasmatic reticulum activity.

From all these observations it is not surprising that Deptor can behave either as an oncogene or oncosuppressor, depending on the cell- or tissue-contexts. This review highlights recent progresses made in our understanding of the many activities of Deptor, describing its transcriptional and post-transcriptional regulation in different cancer cell types. Moreover, here we discuss the possibility of using compounds able to inhibit Deptor or to disrupt its interaction with mTOR as novel approaches for cancer therapy.

## Background

Over the last few years, Deptor (DEP-domain containing mTOR-interacting protein), also known as DEPDC6 (DEP-domain containing protein 6) has become the focus of relevant studies regarding the development and progression of human malignancies, due to discovering its role as a naturally negative regulator of mTOR (mammalian Target Of Rapamicin) [1]. In fact, this protein is an important component of the mTORC1 and mTORC2 complexes and interacts with mTOR, thereby inhibiting its kinase activity [2]. In addition, Deptor and mTOR regulate each other through a negative feed-forward loop [2]. Therefore, Deptor downregulation leads to an increase in mTOR activity, which in turn produces a further reduction of Deptor expression [2].

Deregulation of mTOR is often associated with cancer pathogenesis, and consistent with its inhibitory effect on mTOR activity, Deptor levels are frequently low in most of tumors with few exceptions such multiple myeloma (MM), thyroid carcinoma or lung cancer, where it is found to be highly expressed [1]. These findings highlight the dual role displayed by Deptor in cancer cells, acting either as an oncogene or as a tumor suppressor. Nevertheless, the complete role played by Deptor within cells has not still been completely elucidated.

In this review, we illustrate the role of Deptor as a natural mTOR inhibitor, and its activity in controlling several molecular pathways, such as apoptosis, cell survival, autophagy and endoplasmic reticulum (ER) homeostasis. At the same time, we present a new role of this protein as a transcriptional activator. Furthermore, we describe different molecular ways that regulate its expression at transcriptional and post-transcriptional levels. Finally, we discuss the role of Deptor in several types of tumors and highlight it as a novel therapeutic target to improve the outcome of cancer therapy.

## An overview of Deptor

### Structure of Deptor

Deptor is a monomeric 46 kDa protein encoded by the *DEPTOR* gene located on chromosome 8, in a region of genome, 8q24, rich of several single nucleotide polymorphisms (SNPs), and genes involved in general cancer susceptibility [3].

**Fig. 1:**
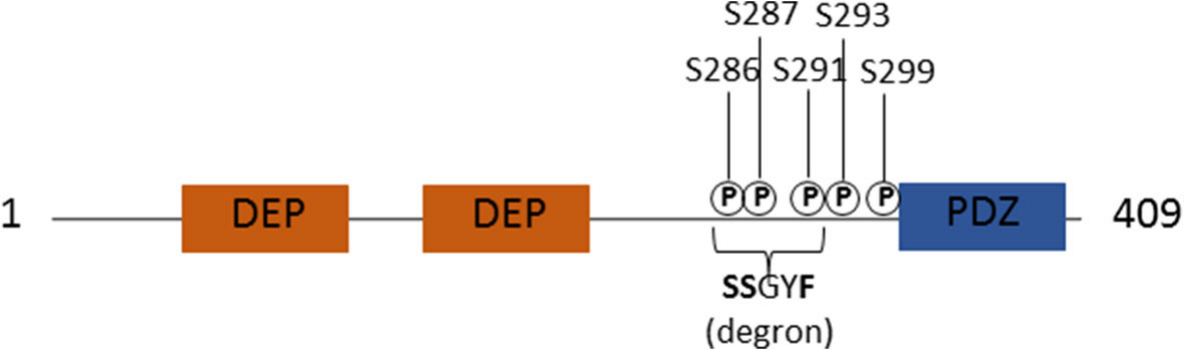
Structure of Deptor. Schematic illustrating Deptor structure: two DEP domains and a PDZ domain. Multiple phosphorylation sites are present between the C-terminal DEP domain and PDZ domain, where resides a consensus binding site, SSGYFS (Deptor degron)

This protein contains 409 aminoacids and it is characterized by the presence of three highly conserved domains: two DEP (Dishevelled, Egl-10, Pleckstrin) domains in tandem, respectively of 84 and 75 aminoacids at the N-terminal region (residues 36–119 and 145–219), which are important for membrane association of signaling proteins, and a PDZ (Postsynaptic density 95, Discs large, Zonula occludens-1) domain of 78 aminoacids at the C-terminal region (residues 330–407), responsible for protein-protein interaction [2, 4] (Fig. 1). A consensus binding site (designed as Deptor degron), SSGYFS, recognized by F box βTrCP for its degradation, is located in the linker region between the C-terminal DEP domain and the PDZ domain. This region also contains multiple phosphorylation sites, which are involved in regulating this protein functions [2, 5] (Fig. 1).

**Fig. 2:**
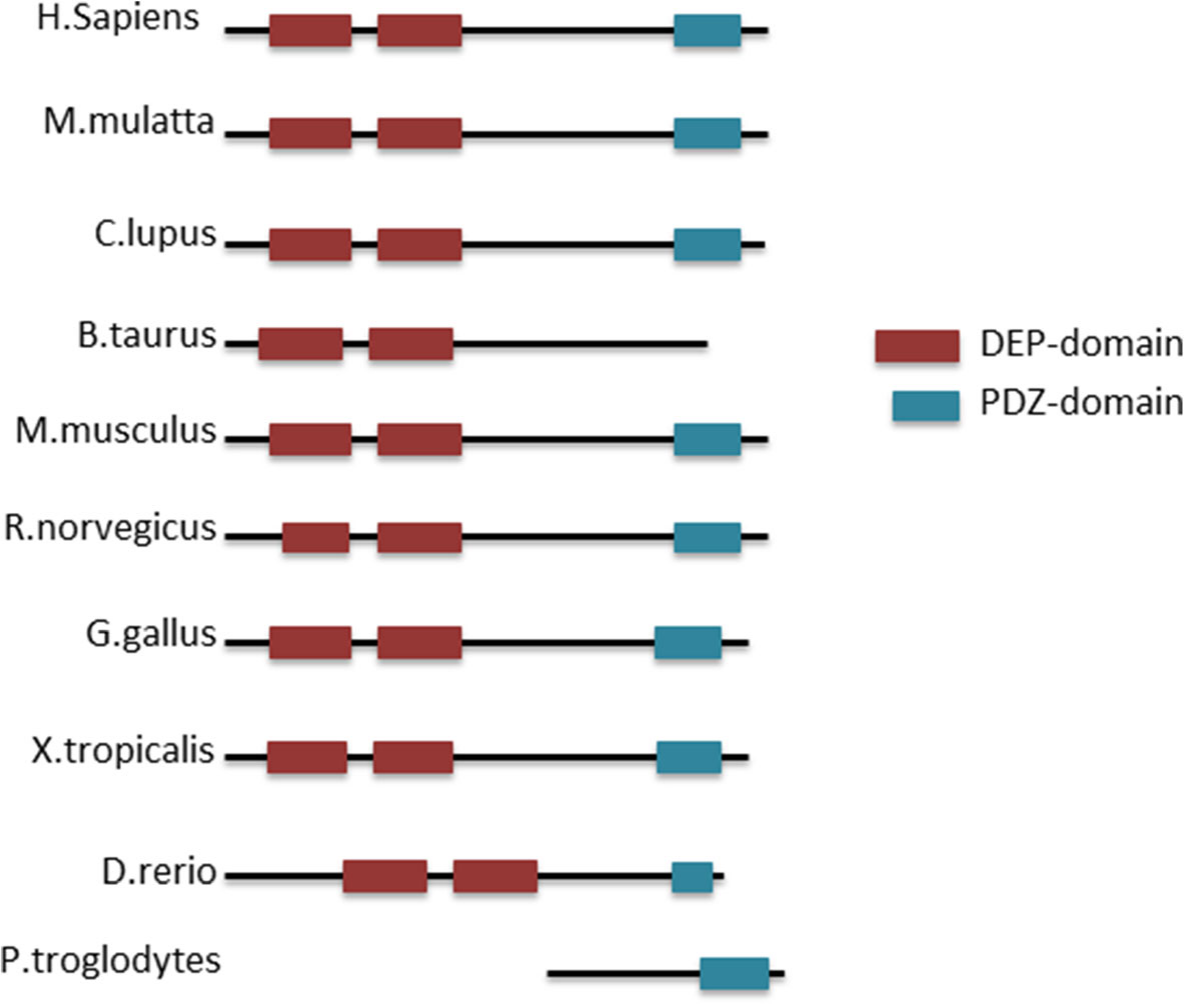
Structure homology of Deptor. Schematic representation of structural features of human Deptor and its orthologues

Deptor gene is found only in vertebrates, sharing an high homology among several organisms [2] (Fig. 2). At least two isoforms of human Deptor produced by alternative splicing, have been described [6]. The isoform 2 lacks of two in-frame exons in the 5’ coding region (residues 42–142) and encodes a shorter transcript of 308 aminoacids [6].

### Deptor expression and localization

High levels of Deptor protein have been described in many tissues, such as muscle tissue and salivary glands, whereas high levels of Deptor mRNA has only been observed in endocrine glands [7]. At the cellular level, Deptor shows cytoplasmic, mitochondrial and nuclear localizations [7–11]. In this regard, the Deptor nuclear localization has been recently associated with its role in gene transcription regulation in MM cells [8].

## Deptor biological functions

### Deptor is a natural mTOR inhibitor

The functional studies regarding Deptor have been essentially focused on its important role as a negative regulator of mTOR kinase activity. Peterson et al. detected Deptor for the first time by mass spectrometry in HeLa cells, and demonstrated its ability to directly bind mTOR within mTORC1 and mTORC2 [2]. mTORC1 and mTORC2 are large complexes composed of six and seven proteins respectively. They share mTOR, mLST8 (mammalian Lethal with Sec-13), Deptor and the Tti1/Tel2 complex [12, 13]. On the contrary, raptor (regulatory-associated protein of mammalian target of rapamycin) and PRAS40 (Proline-Rich Akt Substrate 40 kDa) are specific to mTORC1, while rictor (rapamycin-insensitive companion of mTOR), mSin1 (mammalian Stress-activated map kinase-interacting protein 1) and protor1/2 (protein observed with rictor 1 and 2) are only part of mTORC2 [12, 13]. These two complexes have different functions within the cells. Importantly, mTORC1 is able to respond to intra- and extra-cellular inputs, such as growth factors, oxygen, aminoacids and energy status, and to promote lipid and protein synthesis, by phosphorylating 4EBP1 (eukaryotic translation initiation factor 4E-Binding Protein 1) and S6K1 (ribosomal protein S6Kinase beta-1). Moreover, mTORC1 complex inhibits the autophagy process by phosphorylating and suppressing ULK1 (Unc-51 Like autophagy activating Kinase 1) activity [14]. mTORC2 is much less characterized, but it is known that this complex is involved in cell survival, metabolism and cytoskeletal organization [15]. mTORC2 does not respond to nutrients, but it is sensitive to growth factors like insulin in a PI3K (PhosphatidylInositol-4,5-bisphosphate 3 Kinase)-dependent mechanism [14].

Several findings have showed that Deptor/mTOR interaction produces a strong inhibition of both mTORC1 and mTORC2 activity [2]. Moreover, Deptor and mTOR regulate each other thanks to the existence of a negative feedback loop, in which Deptor downregulation results in an increased of mTOR kinase activity that leads to higher levels of phosphorylation states of the mTORC1/2 substrates, S6K1 and Akt [2].

Since mTOR regulates multiple biological processes involved in mRNA translation, ribosome biogenesis, cell growth and proliferation, autophagy as well as inflammation [12], the ability of Deptor to regulate this kinase have suggested an important role of this protein in the maintenance of cellular homeostasis. In line with this notion, the most relevant functions of Deptor are those involved in proliferation, apoptosis, autophagy, and anti-inflammation responses.

### Cell growth and proliferation

**Fig. 3:**
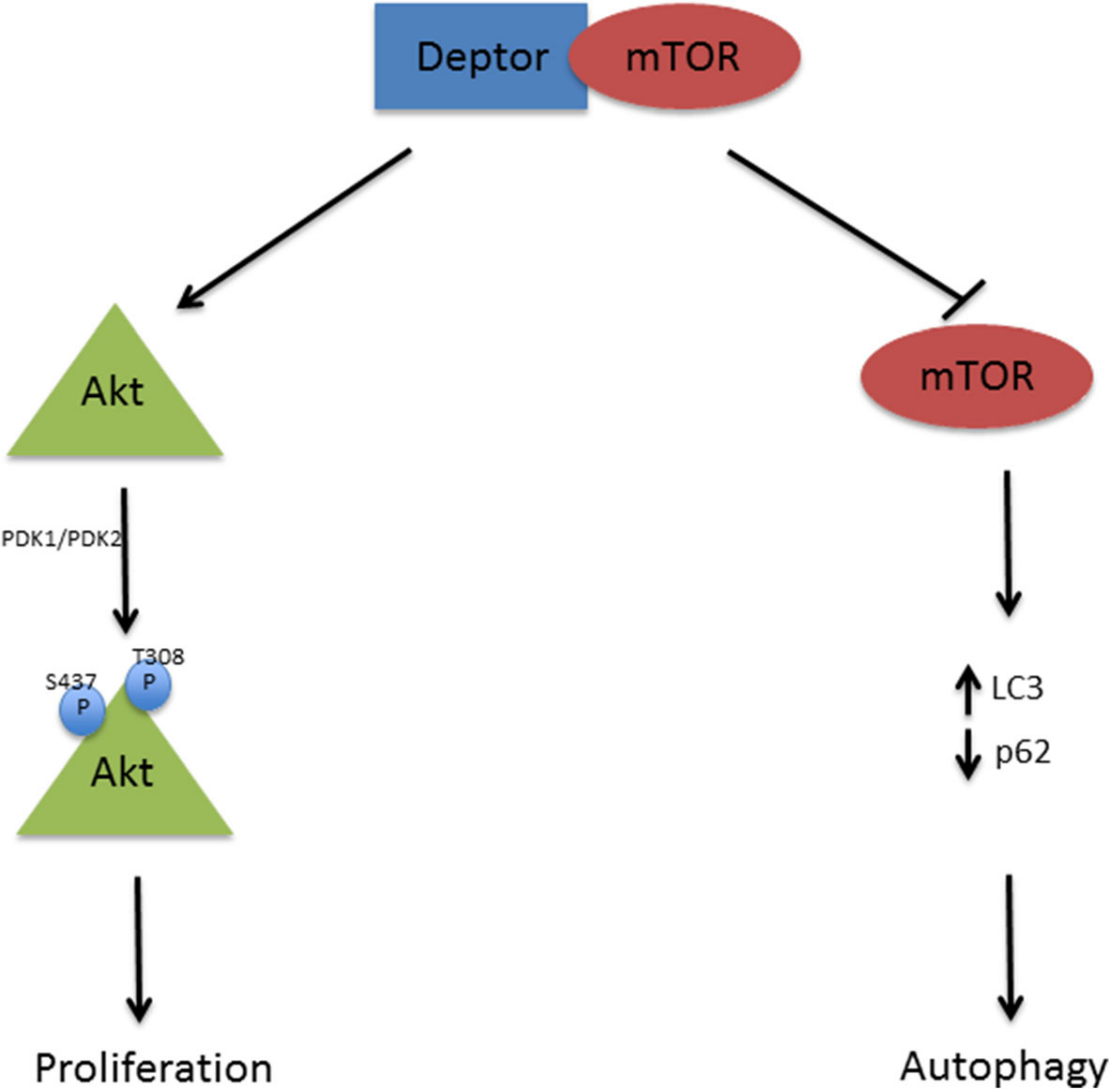
Deptor is a natural mTOR inhibitor. Deptor interacts with and inhibits mTOR kinase activity, leading to the induction of autophagy process. At the same time, it also activates Akt pathway in order to promote cellular proliferation and survival

Many findings support the idea of Deptor being a positive regulator of cell proliferation. As already demonstrated It has been demonstrated that Deptor overexpression is able to inhibit mTORC1 leading to an apparent increase of mTORC2 signaling, inducing Akt phosphorylation at S437 and T308residues (Fig. 3) [2]. This effect seems to be the consequence of the release of the negative feedback signal that normally suppresses PI3K, the major upstream regulator of Akt [2]. PI3K/Akt pathway is frequently hyper-activated in cancer and the activation of its downstream targets is associated with proliferation, differentiation and apoptosis [16]. Indeed, Akt mediates cell survival directly, by inhibiting pro-apoptotic proteins, such as caspase-9 and Bad, and indirectly, by modulating several regulators of cell death including p53 and Nf-KB [17]. In this scenario, high levels of Deptor can sustain cell survival in many cancer cell types.

**Fig. 4:**
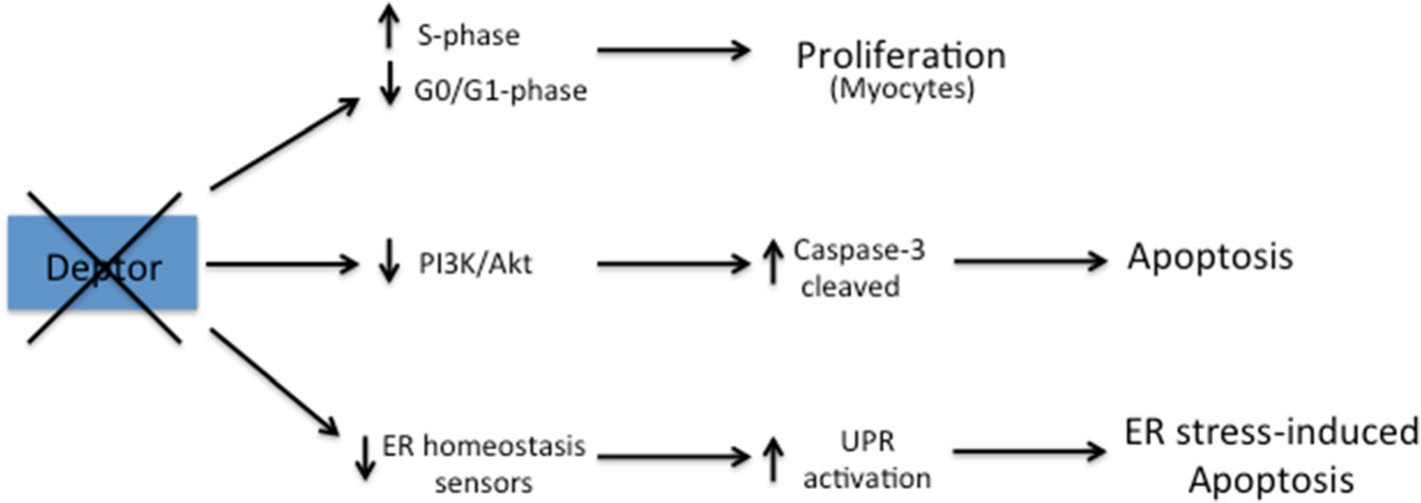
Deptor depletion is associated with different pathway. The outcome of Deptor depletion depends on cellular context. In myocytes, loss of Deptor expression contributes to proliferation, whereas decreased levels of Deptor in other tissues mediate the apoptotic response by activating caspase 3 or enhancing ER stress

On the other hand, Deptor has been found to play an anti-proliferative role in myocytes, where its depletion resulted in an increase of the rate of proliferation [18]. In particular, myoblasts lacking of Deptor expression were larger in diameter, exhibiting also increased cell volume [18]. In addiction, these cells displayed a decreased in the percentage of cells in the G1/G0 phase and concomitantly an increase in the cell number in the active S-phase (Fig. 4) [18].

Interestingly, recent studies have demonstrated a key role of Deptor in the embryonic stem cells (ESCs) differentiation, in both mouse and human [19]. Specifically, high Deptor levels have been found in ESCs, while, they strongly decrease during cellular differentiation [19]. This event suggests a role of Deptor in the maintenance of the pluripotent state of ESCs in association with an increase in mTORC1 activity [19].

### Autophagy

Several studies have reported that Deptor is involved in the activation of the autophagy pathway (Fig. 3). Autophagy is a process of self-degradation by which damaged organelles and misfolded or aggregated proteins are removed by autophagosome degradation [20]. This pathway is important as a barrier to limit tumor initiation, thanks to its ability to prevent oxidative stress and genomic instability [21]. Indeed, this process can be used by cells to balance the sources of energy in response to nutrient stress, such as glucose or serum starvation [20]. Interestingly, Deptor induces the autophagy process by suppressing mTOR kinase activity, a well-known negative regulator of autophagy [5, 22]. In fact, mTORC1 inhibits autophagosome formation, while mTORC2 represses the expression of autophagy related genes (ATG) and other autophagy regulators [23]. In addition, Deptor accumulation due to depletion of βTrCP or glucose deprivation, induces an increase in the level of LC3-II protein and a reduction of p62, well-known autophagy markers (Fig. 3) [5].

In addition, it has been recently demonstrated that Deptor stability is promoted by AMBRA1-mediated Cullin5 inhibition [17]. Moreover, through Deptor accumulation, AMBRA1 establishes a positive feedback loop reinforcing the inhibition of mTOR activity.

Furthermore, two studies have demonstrated an increase of Deptor expression in response to autophagy induction by several kinds of stress [24, 25], and the existence of a correlation between Deptor gene expression and autophagy in MM cells [24]. Of interest, Deptor knockdown has been found associated with a lower expression of autophagy related proteins, such as ATG5 and LC3, supporting the concept that Deptor is a positive regulator of autophagy (Fig. 3) [5, 22]. Nevertheless, the autophagy regulation by Deptor is not limited to its inhibitory action of mTOR, since a recent study have revealed that this kinase may not been directly involved in all steps of this process, being deregulated in the early stages of autophagy and then reactivated in the late stages to facilitate the creation of new lysosomes [26].

An important Deptor role in autophagy has been also highlighted in an Alzheimer’s disease (AD) model [27]. mTOR dysregulation is implicated in AD, since its inhibition plays a key role in protecting against the neurotoxicity induced by the accumulation of the β-amyloid peptide aggregates (Aβ) [27]. In normal condition, mTOR acts as a neuroprotective agent by allowing Aβ clearance via autophagosome. Davies et al. have shown low protein levels of Deptor in precentral gyrus, postcentral gyrus and occipital lobe of a brain with AD and in late-onset AD [27]. This Deptor expression reduction is responsible for an increased of mTOR kinase activity resulting in Aβ accumulation. The Aβ in exceeding, in turn, gave rise to a further reduction of Deptor expression with hyper-activated mTOR ending in enhanced neurodegenerative process [27].

### Apoptosis

Deptor is a potent anti-apoptotic agent. Depletion of Deptor is indeed associated to higher levels of cleaved caspase-3 and PARP (Poly ADP Ribose Polymerase), well-known markers of apoptosis induction (Fig. 4) [2]. In particular, the absence of Deptor is sufficient to trigger caspase-dependent apoptosis [17]. Deptor suppression also induces a reduction in the PI3K/Akt activity, event that prevents the inhibitory phosphorylation of pro-caspase-3, thus promoting the apoptotic process (Fig. 4) [17].

It is still unclear whether this effect on apoptosis is due to a direct effect of Deptor or whether it is the result of the inhibition of mTOR kinase activity [24]. Further studies are therefore necessary in order to explore the existence of some mTOR-independent functions of Deptor in the apoptotic pathways.

### Inflammation

Recently, it has been demonstrated that Deptor is a potent endogenous regulator of endothelial cells (ECs) responses [28]. ECs activation initiates the acute and chronic inflammation through promoting local lymphocyte responses against cell-mediated immune reaction [28]. Several endogenous mechanisms are critical for the prevention of chronic inflammatory diseases and the speed of resolution determines the outcome of an inflammatory reaction. Of note, Deptor has been found expressed in ECs and its depletion resulted in ECs activation with induction of several T-cell chemokines and adhesion molecules [28]. This effect is exerted by affecting mTORC1 signaling, and the activity of ERK1/2 and STAT1 in these cells [28].

Deptor modulation by pro-inflammatory cytokines has been also involved in obesity-associated skeletal muscle inflammation, thus contributing to the onset of insulin resistance and type 2 diabetes [29]. Therefore, Deptor activity would be both a mediator (ECs) and a target (muscle) of inflammatory disorders.

## Deptor is a transcriptional regulator

Most of the studies investigating Deptor have been primarily focused on its role as inhibitor of mTOR kinase activity. Interestingly, the nuclear localization of Deptor in MM cells has been recently observed, leaving to assume that this protein has an additional function in these cells [8]. In particular, Deptor protein has been found accumulated onto the chromatin, thus suggesting its possible involvement in transcriptional regulation. Consistent with these findings, a RNA-seq analysis of MM cells depleted for Deptor expression have revealed approximately 2000 transcripts modulated by this protein [8]. Gene Ontology analysis of these differentially expressed genes have shown that Deptor is able to regulate several pathways involved in protein localization or transport, and in particular in ER homeostasis. These data have been confirmed by the analysis of microarray public data of MM patients, in which a significant correlation between Deptor and several ER homeostasis genes, such as ERLIN2, KEAP1, PSEN2 and DERL3, has been found [8]. Moreover, Deptor has been detected on specific promoter regions of ER homeostasis genes, thus confirming its direct involvement in gene transcription [8]. MM cells are characterized by a high demand of protein synthesis to produce and secrete large amounts of immunoglobulins (Igs), thus resulting in high levels of ER stress [30]. For this reason, MM cells evolved a way to survive ER stress by activating Unfolded Protein Response (UPR) [30]. Interestingly, Deptor depletion produces a further increase in ER stress in MM cells, thus inducing apoptotic pathway [8]. In particular, Deptor-depleted MM cells have shown increased UPR activation, triggering a signaling cascade resulting in elevated apoptosis rate (Fig. 4). Therefore, high levels of Deptor in MM cells contribute to keeping ER homeostasis under control by inhibiting apoptosis-induced ER stress [8]. The overall treatment of MM patients is based on the use of proteasome inhibitor (PI) Bortezomib (Bz), which is able to activate apoptosis by various ways [31]. Notably, Deptor depletion made MM cells more responsive to cell death induced by Bz treatment [8].

## Deptor regulation

Several functional studies have demonstrated that Deptor expression is subject to a tight regulation exerted by many mechanisms. Peterson et al. have showed that both mTORC1 and mTORC2 complexes negatively regulate Deptor protein and mRNA expression [2]. These findings might explain the inverse correlation between the high activity of mTORC1/2 complexes and low levels of Deptor. However, Deptor regulation resulted more complicated, involving post-transcriptional and transcriptional mechanisms mediated by both complexes. Indeed, it has been demonstrated the existence of an interplay between ßTrCP-Deptor-mTOR in the regulation of cell survival and autophagy in response to environmental changes [5]. In particular, in a low-energy state, Deptor binds mTOR and inhibits its kinase activity, whereas in a high-energy state, mTOR rapidly phosphorylates Deptor at the βTrCp binding degron, mediating its degradation by SCF E3 ligase [5, 22, 32]. Specifically, upon serum stimulation, mTOR phosphorylates Deptor at S293 and S299 residues promoting in such way its phosphorylation by S6K1 and RSK1 (Ribosomal S6 Kinase 1) kinase on the three serine residues (S286, S287, S291) contained in the degron site (SSGYFS), which becomes recognizable for ßTrCP binding [5, 22, 32]. ßTrCP is a F-box protein that forms, in association with SKP1 (S-phase Kinase-associated Protein 1), cullins, and a RING protein, RBX (RING BoX protein), the E3 ubiquitin ligase complex SCF (Skp1-Cul1-F-box) [33].

In addition, another mechanism has been described illustrating how mTOR can regulate Deptor expression independently of its phosphorylation [34]. Indeed, in response to mitogenic stimulation, Phosphatidic Acid (PA), generated by PLD1 (PhosphoLipaseD1), binds mTOR, displacing Deptor from mTORC1, and consequently activating it [34].

Unlike protein stability, which is regulated by phosphorylation and consequently ubiquitination by E3 ligase, the transcriptional regulation of Deptor is more complicated and depends on the cellular context.

In a recent study, Deptor expression has been found modulated by Che-1/AATF (Che-1) protein in response to several stress condition [25]. Che-1 is emerging as an important protein that binds the RNA polymerase II, and it is involved in a wide range of cellular processes, such as proliferation, cell cycle control and apoptosis [35]. Moreover, Che-1 plays an important role in DNA damage response [36]. For instance, in response to DNA stress, Che-1 is phosphorylated by ATM/Chk2 kinases and is recruited on the p53 promoter thus inducing its expression and the expression of p53-dependent apoptotic genes [36].

Previous studies have showed that mTOR activity is subjected to several external stimuli, such as nutrients and energy sensors [14]. In addition, dysregulation of the mTOR signaling cascade is associated with many types of stress, such as DNA damage, glucose or oxygen deprivation [25]. In agreement with these observations, it has been demonstrated that upon these kinds of stress, Che-1 is able to negatively regulate mTOR kinase activity, by inducing Deptor expression [25]. In particular, in response to stress, Che-1 is phosphorylated and recruited onto Deptor promoter, allowing its expression and leading to mTOR inhibition [25]. In agreement with a transcriptional regulation of Deptor by Che-1, a positive correlation between these proteins was observed during MM progression [25].

An analysis of several databases containing transcriptional profiles of human tumors and cancer cell lines have revealed Deptor downregulation in most cancer types compared to normal tissue, with the exception of MM, where this gene has been found upregulated [2]. Many MMs expressing high levels of Deptor are characterized by Ig gene translocation of c-MAF and MAFB transcription factors, resulting in elevated levels of these proteins. Deptor has been found to be a direct target of these factors, and in agreement with this evidence, a transcriptional profiling study revealed the induction of Deptor expression by MAFB overexpression. Moreover, c-MAF depletion produced a reduction of mRNA and protein levels of Deptor in MM cells [2].

More recently, mechanistic insight on Deptor regulation has been provided in T-cell leukemia (T-ALL), revealing its crucial role in leukemogenesis [37]. Notably, in these cells Deptor expression is strongly upregulated by the oncogene NOTCH1, contributing in such way to the activation of Akt, a critical effector of the T-ALL pathogenesis [37]. Deptor and NOTCH1 show a positive correlation in primary T-ALL samples, and Deptor-ablated T-ALL cells display a reduction of cellular proliferation and an increase in cell death [37]. However, prolonged Deptor depletion in these cells results in diminished death-promoting effect, suggesting the possibility that T-ALL cells may acquire resistance through alternative mechanisms [37].

Finally, two transcriptional cofactors, Six4 and Baf60c, have been found to stimulate Deptor expression by increasing its transcription in cultured myotubes [29]. In particular, Baf60c in the muscle tissue promotes glycolytic metabolism by upregulating Deptor expression [29]. In rodent models of obesity, the expression of these proteins is reduced because of repressive epigenetic modifications of their promoters due to pro-inflammatory cytokines [29]. In line with these findings, Baf60c/Deptor pathway could be considered a link between skeletal muscle inflammation and glucose homeostasis in obesity [29].

## Deptor and cancer

Deregulation of mTOR signaling is associated with aging and development of human diseases, including cancer, obesity, type 2 diabetes and neurodegeneration [14]. In accordance with these observations, several studies have highlighted an important role of Deptor in the development and in the maintenance of many of these diseases. Nevertheless, the exact role of Deptor in cancer still remains controversial, due to its dual behaviour either as an oncogene or as a tumor-suppressor, depending on the cellular and tissue context (Table 1).

**Table 1:** The dual role of Deptor in cancer

Tumor suppressor role	Oncogene role
Lung Adenocarcinoma [11]	Multiple Myeloma (MM) [2]
Pancreatic ductal adenocarcinoma (PDAC) [10]	Differentiated thyroid carcinoma (DTC) [45]
Liver cancer [38]	Cervical squamous cell carcinoma (SCC) [44]
Colorectal cancer [39]	Triple-negative breast cancers (TNBCs) [46]
Triple-negative breast cancers (TNBCs) [46]	

In several tumor models in which mTOR is massively activated, Deptor levels are much lower than corresponding normal tissue [2]. In colorectal, liver and pancreatic cancer [38, 39] and in a subset of tumors like MM, Deptor mainly acts as a tumor suppressor, since its loss promotes growth and survival by activating Akt/mTOR signaling, resulting in phosphorylation of SGK1 (Serum and Glucocorticoid regulated Kinase 1) and of its substrate NDRG1 (N-Myc Downstream Regulated 1) [40, 41].

Deptor has been found to play an important role in EGFR (Epidermal Growth Factor Receptor)-induced tumor progression of lung adenocarcinoma and its expression is significantly reduced in patients with this type of pathology [11]. In lung adenocarcinoma where EGFR is overexpressed, low levels of Deptor are observed with a consequent enhanced mTOR kinase activity. Furthermore, Deptor downregulation leads to tumor progression and drug resistance. More importantly, upon treatment with gefitinib, an EGFR tyrosine kinase inhibitor, it has been observed an increase in Deptor expression, providing relevant evidence on the importance of Deptor in the development of new therapeutic options [11].

A remarkable oncosuppressor role played by Deptor is observed in the pancreatic ductal adenocarcinoma (PDAC) [10], where low expression of this protein is detected in pancreatic pre-neoplastic lesions, and gradually lost in 99% of PDAC tissue. In agreement, ectopic overexpression of Deptor in these cells blocked cell growth and apoptotic cell death [10]. It is still unclear what the exact mechanism is by which these effects are produced. Probably, Deptor overexpression counteracts the mTOR activity, but it is also possible that other mechanisms may play an important role [10]. The evidence that Deptor loss promotes initiation and progression of pancreatic tumorigenesis reinforces this hypothesis [10].

On the contrary, in a specific subset of MM (non-hyperdiploid type and with c-MAF or MAF-B translocation) and thyroid carcinoma, Deptor behaves like an oncogene, as its overexpression results in mTORC1 activity inhibition and a contemporary Akt activation, promoting cancer cells survival [2, 42, 43]. Interestingly, all these tissues are characterized by a significant protein production and secretion, resulting in an elevated ER stress. Therefore, In this context high levels of Deptor could be required for sustaining ER functions and allowing cell survival.

An oncogenic role of Deptor has also been reported in cervical squamous cell carcinoma (SCC) where Deptor overexpression sustains the PI3K/Akt activation required for cell survival and proliferation [44]. In agreement, Deptor depletion leads to apoptotic decision in these cells, through upregulation of p38 MAP kinase, responsible for the activation of apoptosis induced by p53 and PUMA proteins [44].

Similar findings have been reported in differentiated thyroid carcinoma (DTC) cell lines and tissues, where Deptor levels have been found associated with lymph node status, extra thyroid extension and distant metastasis, confirming its important role in tumor progression [45]. However, Deptor mRNA did not correlate to protein levels in these cells, suggesting the presence of post-transcriptional events controlling Deptor degradation [45]. In agreement, a strong correlation between Deptor overexpression and reduced overall survival has been observed in DTC patients [45].

The role of Deptor in breast cancer however, remains to be fully elucidated. Indeed, Parvani et al. demonstrated Deptor dual behaviour during the development and metastatic progression of triple-negative breast cancers (TNBCs) [46]. In particular, low levels of Deptor in primary tumors are required for the acquisition of EMT (epithelial mesenchymal transition) and invasive phenotypes in association with cell cycle arrest. In stark contrast, its upregulation in metastatic lesions participates to the induction of anti-apoptotic and chemoresistant activities thanks to its ability to regulate survivin expression [46].

## Deptor as a new attractive molecular target

The findings described above describe an important role of Deptor in several molecular pathways, often dysregulated in cancer. As already mentioned, Deptor is highly expressed in some cancer types, such as MM and T-ALL, and it is associated with poor prognosis and reduced survival for patients. Consistent with these observations, Deptor depletion in these cells resulted in apoptotic cell decision. Therefore, it is conceivable that the pharmacologically reduction of Deptor may have therapeutic benefits for the MM treatment in the future.

The studies carried out so far showed how Deptor depletion could be of help in the improving the outcome of the MM treatment. In fact, Deptor depletion in combination with different drugs, such as Bz and melphalan, increases MM cells sensitivity to treatment, promoting an augment of apoptotic rate [8, 47]. Interestingly, Deptor over-expression may be considered as a predictive biomarker for therapeutic response in MM patients treated with thalidomide [48].

In addition, a recent study demonstrated a Deptor involvement in the suppressing effect of metformin on mTOR signaling and on cell proliferation in human liver cancer cells [38]. Specifically, metformin treatment leads to increased Deptor protein levels, by its inhibitory effect on proteasome activity [38].

Moreover, since Deptor functions are mainly based on its interaction with mTOR kinase, it is conceivable that the pharmacological disruption of Deptor-mTOR interaction might have therapeutic benefits for MM treatment in the future. In line with this notion, it was recently demonstrated that MM cells expressing Deptor are sensitive to NSC12640 [49]. This compound acts by binding to Deptor at PDZ domain, thus preventing the interaction with mTOR [49]. The disruption of mTOR-Deptor interaction allowed the activation of mTORC1/2 complexes and the induction of apoptosis [49]. The cytotoxicity induced by NSC12640 is directly correlated with Deptor protein expression, and is mediated by the activation of mTORC1 and induction of p21 expression. In agreement, previous studies have revealed that Deptor knockdown induces p21 expression in a p53 independent manner, with a concomitant inhibition of MM cells growth [50].

As reported in other studies, a rare subtype of myeloma, IgD MM, that represents less than 2% of all myeloma, is associated with aggressive course, resistance to chemotherapy and poor outcome [51]. Thanks to the use of conventional chemotherapy, autologous stem cell transplantation and the introduction of new drugs, such as Bz, an increase in overall survival was achieved [51]. Could be of interest to monitor the Deptor protein levels in this MM isotype and verify whether modulating Deptor expression it would be possible to improve the outcome for IgD MM patients.

Nevertheless, further efforts will be necessary to fully understand the exact mechanism of action of Deptor and to identify all the pathways implicated in its regulation, in order to unveil new possible anticancer therapeutic approaches.

## Conclusions

As described in this review, Deptor appears to play an important role in the pathogenesis of many types of cancers, mainly through its role in controlling the activity of mTOR. However, recent studies have also revealed other its possible functions within the cell, making it even more important research on how it can become a target of anticancer therapies.

## Abbreviations

AD: Alzheimer’s disease
Bz: Bortezomib
DTC: Differentiated thyroid carcinoma
EC: Endothelial cell
EMT: Epithelial mesenchymal transition
ER: Endoplasmic Reticulum
ESC: Embryonic stem cell
Ig: Immunoglobulins
MEC: Mammary epithelial cells
MM: Multiple myeloma
PDAC: Pancreatic ductal adenocarcinoma
PI: Proteasome inhibitor
SNP: Single nucleotide polimorfism
T-ALL: T-cell leukemia
TNBC: Triple-negative breast cancer
UPR: Unfolded protein response
